# Temporomandibular Joint Disorders in Females with Adolescent Idiopathic Scoliosis: Long-Term Effects of Milwaukee Brace Treatment

**DOI:** 10.3390/jcm11061721

**Published:** 2022-03-20

**Authors:** Jakub Glowacki, Joanna Latuszewska, Adam Okret, Natalia Skowron, Ewa Misterska, Justyna Opydo-Szymaczek

**Affiliations:** 1 Department of General Orthopaedics, Musculoskeletal Oncology and Trauma Surgery, Poznan University of Medical Sciences, 61-701 Poznan, Poland; 2 The Faculty of Educational Studies, Kazimiera Milanowska College of Education and Therapy, 61-473 Poznan, Poland; j.latuszewska@wp.pl; 3 Department of Spondyloortopaedics and Biomechanics of the Spine, Poznan University of Medical Sciences, 61-701 Poznan, Poland; adamokret@poczta.onet.pl; 4 Center for Early Intervention of the Polish Association for People with Intellectual Disabilities in Poznan, 61-446 Poznan, Poland; natalia.skw@vp.pl; 5 Department of Pedagogy and Psychology, University of Security, 60-778 Poznan, Poland; emisterska1@wp.pl; 6 Department of Pediatric Dentistry, Poznan University of Medical Sciences, 61-701 Poznan, Poland; jopydo@ump.edu.pl

**Keywords:** scoliosis, brace treatment, temporomandibular joint disorders

## Abstract

Patients with adolescent idiopathic scoliosis (AIS) more frequently suffer dysfunctions of dento-skeletal complex. To our knowledge, no study has ever evaluated the temporomandibular joint disorders (TMD) of AIS patients at least 23 years after the completion of Milwaukee brace treatment. We aimed to provide a complex assessment of TMD and AIS patients treated with a Milwaukee brace, in a minimum 23-year follow-up, using radiological, clinical, and socio-demographical data, and to adapt the TMD Disability Index Questionnaire (TMDQ) and Fonseca’s questionnaire (FQ) to Polish conditions. In total, 42 healthy females and 30 AIS patients with a minimum of 23 years after a completed Milwaukee brace treatment were asked to complete the Polish version of (TMDQ-PL) and (FQ-PL). AIS patients present higher TMD levels than healthy controls. Significant differences exist between TMDQ-PL and FQ-PL (both in total scores and particular sub-sections), and AIS patients. Clinical and radiological factors affected the TMDQ-PL and FQ-PL results. Adult patients with scoliosis treated conservatively present limitations in everyday activities connected with the temporomandibular joint (TMJ). The variety of curve-related factors in a long-term follow-up of wearing the Milwaukee brace influence TMJ.

## 1. Introduction

In the 60s and 70s, extensive studies investigated the effects and consequences of using the Milwaukee brace in scoliotic patients on TMJ. Most studies were carried out on dental occlusion, growth of the dento-skeletal complex, and teeth position in patients with adolescent idiopathic scoliosis (AIS) [1,2,3,4]. Especially, longitudinal observational analyses, case reports, and case-control reports were reported [1,2,3,4].

Since then, the Milwaukee brace has been modified to avoid dento-skeletal abnormalities [5,6]. Notwithstanding, recently published studies have concluded that children and adolescents with AIS are more likely to suffer malocclusions, dentofacial asymmetry in the sagittal and vertical dimensions, shift in position of the upper and the lower dental arches, and deviations of crossbite [7,8]. Researchers hypothesized that compensatory aberrance of the cervicocephalic posture provokes a tendency for head tilt and rotation. Head posture has been found to be strongly correlated to craniofacial morphology [5,6,7,8]. It must be emphasized that frequent occurrence of dentofacial deviations in AIS indicates a need for orthodontist and orthopedist cooperation at early stages of diagnosis and treatment.

As mentioned before, a majority of studies have analyzed the link between dento-skeletal abnormalities and patients treated with a Milwaukee brace. However, most of them have focused on dental occlusion, growth of the dento-skeletal complex, and teeth position. Very little has been said about scoliosis effect and previous treatment on TMD [9,10,11]. No report, to our knowledge, has investigated TMD related to wearing a Milwaukee brace in adults treated for AIS in puberty. This is the first study of patients at least 23 years after completing the Milwaukee brace treatment, involving an assessment of TMD and comparison with a group of healthy controls.

There is an abundance of information in the literature about adult patients with AIS in long-term follow-up studies [12,13,14,15,16,17,18,19,20,21]. However, according to our knowledge, none of the studies have investigated TMD related to wearing a Milwaukee brace in adults treated in puberty for AIS.

Therefore, this study was designed to assess the effects of the Milwaukee brace on TMD in adult AIS female patients, in a minimum 23-year follow-up, in relation to the non-scoliotic control group. We aimed to establish associations between TMD and radiological, clinical, and socio-demographic data. As there is no Polish version of the TMDQ and FQ, the second aim of the study was to adapt those assessment tools to Polish cultural conditions and verify their psychometric properties on a group of adult patients treated with a Milwaukee brace in adolescence. Our study is an attempt to achieve these aims.

First, we hypothesized the presence of differences between scoliotic and non-scoliotic groups in terms of socio-demographic characteristics, TMD.

Second, we hypothesized that significant relationships between TMD, daily life activities, and radiological, clinical, and brace treatment-related variables would be discovered.

Finally, we hypothesized that if we adapt the TMDQ and FQ to Polish cultural conditions, we will have tools that are equivalent to the original English-language method.

## 2. Materials and Methods

### 2.1. Structure of the Study

**Figure 1:**
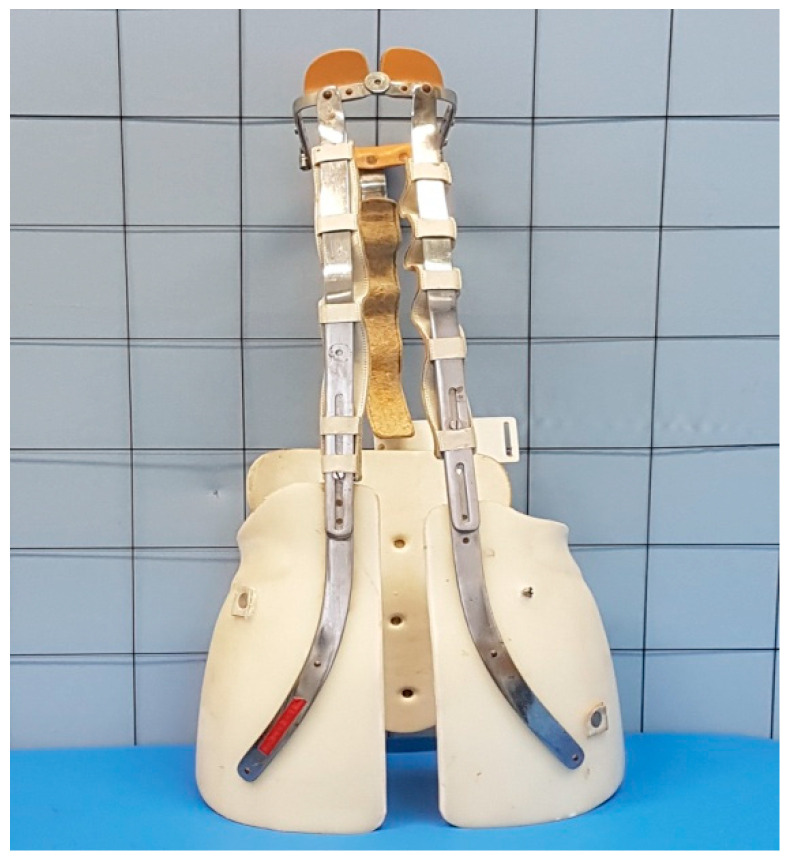
The Milwaukee brace.

**Figure 2:**
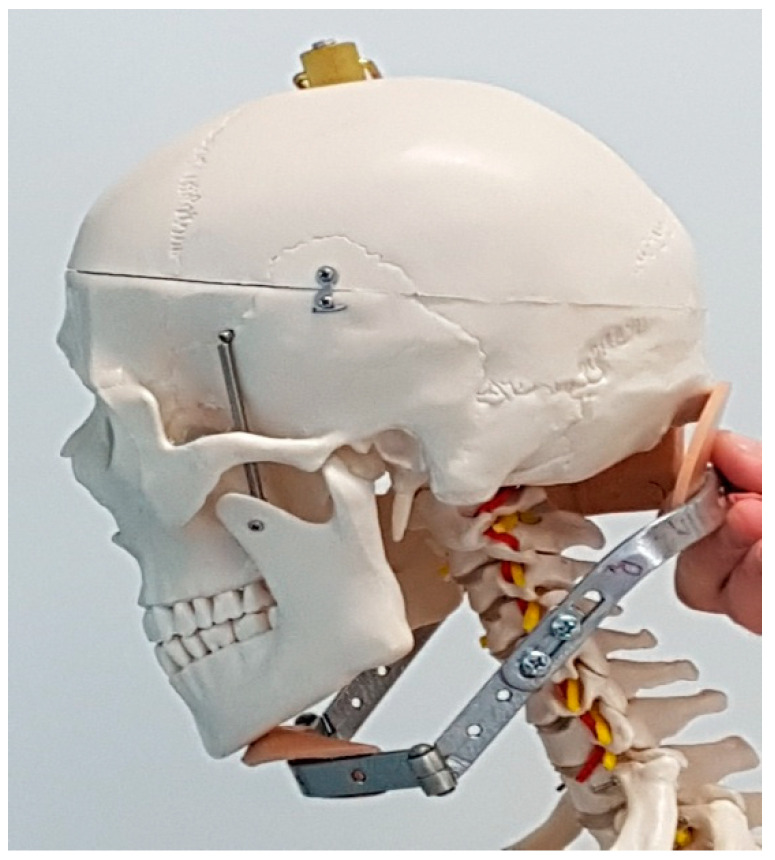
Mandibular and occipital pads.

In the study, results concerning the implications of brace treatment in adult AIS females (scoliosis group-SG) treated with a Milwaukee brace were evaluated. Based on an extensive search of Pediatric Orthopedics and Traumatology Clinic charts, the orthopaedic surgeon retrospectively reviewed the clinical records and radiographs of all female patients who had successfully completed a course of treatment with Milwaukee orthosis between 1974 and 1990. This brace was constructed of the pelvic girdle, vertical metal bars, and neck ring with mandibular and occipital pads (see Figure 1 and Figure 2)

Forty patients met the criteria for inclusion, but due to a change in personal details (such as address or family name after marriage), not all of them were contacted. Finally, 30 women participated in the evaluation.

A control group of healthy females (healthy controls group-HG) was selected for comparison based on random sample choice. The study and control groups were tested for equivalence in regard to their size, and quantitative and qualitative characteristics.

The groups were interviewed for age, work, marital status, number of children and how they were delivered, rate of caesarian sections and complications during delivery, place of residence, and active hobbies. All study participants were examined using the same protocol, except for the radiological evaluation performed in scoliosis patients only. They were informed in detail about the objective of the study. They understood that they would be anonymous, and that their personal information would not be disclosed. All participants signed written informed consent to participate in the study. The study design was approved by the Bioethics Commission of Poznan University of Medical Sciences No. 528/13, and carried out in accordance with the Declaration of Helsinki.

### 2.2. Clinical and Radiological Examination

Clinical and radiological examinations were performed by experienced orthopedic surgeons at three time points: before and after completed treatment with Milwaukee brace, and then in the current follow-up, and were taken in an upright position with the iliac ala exposed in an anterior–posterior projection. Data concerning the former treatment regimens and radiological findings were gathered from a chart and radiograph review. The physical examinations were performed by AO, the third study author, and radiographic measurements were conducted by JG, the first study author. All participants were clinically examined by an experienced dentist, JO, the sixth study author. The dental evaluation did not record any posterior teeth loss.

### 2.3. Patient Sample

Thirty AIS patients with a minimum of 23 years after completed Milwaukee brace treatment were included in the study. All treatments were completed before the patients reached 19 years of age. In all cases, the scoliosis was not detected before 10 years of age, and was not combined with any major spine deformities at the time when brace treatment was implemented. The following exclusion criteria were applied during clinical examination: neurological disorders within the head and neck area, neoplastic diseases, trauma and previous surgical treatment in the head and neck area in the 6 months before the examination, any inflammatory conditions in oral area, illness or injury in the cervical spine area, orthodontic treatment, possession of dental prostheses (regardless of type), and pregnancy.

### 2.4. Healthy Controls

The control group consisted of 42 females, matching the age profile of the patient groups. Inclusion criteria used in the study were: female gender, four support zones of dental arch, complete dentition, and very good physical health. The exclusion criteria for the control group were: previous back surgery or significant scoliosis, which was ruled out by an experienced orthopedic surgeon during clinical examination that included the use of Perdriolli’s scoliometer. None of the controls had a trunk rotation of more than 5°, according to Danielsson et al. [12].

### 2.5. Questionnaires Used in This Study

The TMJ function was assessed using the TMD Disability Index Questionnaire and Fonseca’s questionnaire.

The TMD Disability Index Questionnaire subjectively investigates TMD symptoms and related limitations to TMJ function during everyday activities [22]. The questionnaire is the first component of The Steigerwald/Maher TMD Disability Questionnaire, which is based on the Oswestry Back Pain Questionnaire and the Neck Pain and Disability Questionnaire (Vernon Minor) [23]. The TMD Disability Index Questionnaire contains 10 statements, including specialized functions of the TMJ such as talking, dental care, eating, and social activations, and non-specialized functions of the TMJ. Functional limitation is measured on a scale of 0 to 4, where 0 corresponds to no limitation and 4 indicates maximum restrictions. The minimum sum of points was 0 and the maximum was 40. A higher score is indicative of greater levels of disability. The general result is presented in percentage values [24]. Fonseca’s Questionnaire is a modified version of Helkimo’s index, and provides a multidimensional assessment to classify TMD severity.

The psychometric properties of Fonseca’s Questionnaire have been established. Fonseca’s Questionnaire obtained 95% reliability and correlation with the Helkimo’s index (r = 0.6169, *p* < 0.05) [25,26]. The questionnaire consists of two main parts. The first part focuses on demographic information and past medical, dental, TMJ, and facial trauma histories. The second part is composed of 10 questions regarding the presence of TMJ pain, head and neck pain, pain while chewing, questions on parafunctional habits, limitation of joint movement, perception of malocclusion, and emotional stress. Each of the responses is selected as “yes”, “sometimes”, and “no”, which are assigned values of “10”, “5”, and “0”, respectively. The maximum possible score is 100. Interpretation based on the obtained results indicates that the higher the score, the more severe the TMD. The following subdivision of the total score is as proposed: 0–15 points are interpreted as without dysfunction, 20–40 points indicate light dysfunction, 45–60 points mean moderate dysfunction, and 70–100 points indicate severe dysfunction [27,28].

### 2.6. Translation and Adaptation Procedure

The process of cross-cultural adaptation of the TMD-Q and FQ for Polish settings was compliant with guidelines proposed by Beaton et al. [29], and comprised a number of stages. In the first stage, two translators working independently translated the English versions of the TMD-Q and FQ into Polish. Polish was the native language of these translators. One of the translators, who had a medical background, was instructed on the whole process of the adaptation. The other translator had no medical experience, and received no information on the project. In the second stage, these translations were compared and synthesized into a single version by the two translators and the project’s authors. The third stage consisted of the so-called reversed translation. Two independent translators, both native speakers of English, translated a compromised version of the Polish translation into the language of the original document. The translators were not familiar with the original language versions.

The objective of this stage was to assure the equivalence of the two versions and to identify possible mistranslations. In the final step, a committee of translators, an orthopedic surgeon, a statistician, and a psychologist reviewed all the translations to create a pre-final version of the questionnaire. Then, 30 patients filled out the Polish language versions of TMDQ-PL and FQ-PL twice with a two-day interval. Considering the course of the translation process, the questionnaire items were translated easily, with some grammar discrepancies due to different linguistic backgrounds.

Most of the subjects understood the translated items well and did not report difficulties during completion.

The following tests on the psychometric properties of TMDQ-PL and FQ-PL were conducted:

We analyzed means, minimal and maximal values, standard deviations, and 95% confidence intervals for the general results of TMDQ-PL and FQ-PL and for individual TMDQ-PL questions. To assess the internal consistency of TMDQ-PL and FQ-PL, Cronbach’s alpha was calculated. Cronbach’s alpha values were accepted as follows: 0.80 as excellent, 0.70–0.79 as adequate, and <0.70 as poor [30]. Concerning content validity, we analyzed floor and ceiling effects (% of patients with the minimum score and % of patients with the maximum score). Ceiling and floor effects were considered to be present if more than 15% of respondents achieved the lowest or highest possible total score [31]. Test–retest reliability was calculated using the Intra-Class Correlation (ICC) as an alternative to Pearson’s product moment correlation. Values of ICC above 0.80 were considered as evidence of excellent reliability [27].

### 2.7. Missing Data

The Polish sample was complete; therefore, all calculations were computed with complete cases only.

### 2.8. Statistics

Concerning the quantitative statistical features, e.g., age, apical translation, Cobb angle, and questionnaire results, we determined the mean, 95% confidence intervals, range, and standard deviations. Regarding qualitative features, e.g., curve type, educational level, marital status, or place of residence, we provided the number of units that belong to the described categories of a given feature and their respective percentages. The chi-square test was used to determine if the investigated sample sizes were equivalent. The chi-square test was used to compare qualitative features between persons with scoliosis and healthy controls. The Mann–Whitney test was utilized to compare differences between both groups in regard to quantitative characteristics. We used Spearman’s rank correlation (marked as rS) to establish relations between quantitative data, such as age, duration of brace application, apical translation, and Cobb angle, and questionnaire results. To determine dependency between quantitative and qualitative characteristics, e.g., between questionnaire numerical data and marital status, place of residence, and curve type, the ANOVA Kruskal–Wallis test was used. To protect against Type I errors, a Bonferroni adjustment for multiple comparisons was made in a way that the accepted alpha level (*p* = 0.05) was divided by the number of tests conducted in each section. As the border level of statistical significance, we adopted *p* = 0.05; test results whose p-value exceeded this level were treated as insignificant. For test results whose p-value did not exceed the level of 0.05, effect size (ES) was calculated by means of Cramer’s V or Glass’s Δ. Statistical calculations were performed through Statistica software.

## 3. Results

### 3.1. Clinical and Radiological Data

The sample sizes are equivalent (*p* = 0.157). The patients’ mean follow-up period was 27.77 yrs. SD 3.30 (range 23–35 years). The Milwaukee brace was worn for a mean of 22.9 hrs. daily SD 0.31 (range 22–23). The average length of brace application was 45.47 months SD 20.00 (range 24–104).

In accordance with the criteria of the Scoliosis Research Society regarding the location of the apex [28], thoracic scoliosis was identified in 21 patients (70%), thoracolumbar in 2 patients (6.67%), and lumbar curves in 7 AIS patients (23.33%). Five patients (16.67%) qualified for scoliosis surgery after a completed brace treatment, but refused to undergo an operation. The curve change from the end of treatment to the present study was 9.1 angles SD 7.64 (range 0–27). For additional clinical and radiological characteristics of the patient group, see Table 1.

### 3.2. Socio-Demographic Data

The mean age of patients (SG–study group) at the follow-up was 41.13 yrs. SD 3.87 (range 35–55), whereas the mean age of controls (HG–healthy group) was 42.05 yrs. SD 7.41 (range 22–61). Twenty-eight females with AIS (93.4%) and 29 healthy controls (69%) were married (for additional data, see Table 2).

### 3.3. Distribution of the Results

Mean scores and standard deviations, the minimum, maximum, and 95% confidence intervals, were calculated for both administrations of the TMDQ-PL and FQ-PL. In the patients’ sample, the mean value of the TMDQ-PL total score equaled 4.27 (SD 4.89) in the test and 3.23 (SD 3.86) in the retest. The mean level of disability in the test equaled 11.0% (SD 12.0), and in the retest 8.0% (SD 9.0), which is interpreted as a normal function of TMJ. The mean value of the FQ-PL total score was 32.67 (SD 19.94) in the test, and 32 (SD 22.15) in the retest.

The highest score, indicating the most disturbed function of TMJ in scoliosis, was obtained for question 10 of the TMDQ-PL, concerning dizziness both in the test (0.83 SD 0.91) and in the retest (0.80 SD 0.80) (see Table 3).

The lowest score of TMDQ-PL in scoliosis patients was obtained for question 2 (concerning brushing teeth/flossing) in the test, which was 0.13 (SD 0.43), and in the retest, which was 0.07 (SD 0.25) (see Table 3).

### 3.4. Internal Consistency

The internal consistency (coefficient Alpha) of the TMDQ-PL was excellent, and equaled 0.87 (95% CI from 0.79 to 0.93) in the test and 0.87 (95% CI from 0.78 to 0.93) in the retest. Similarly, Cronbach’s alpha of the FQ-PL was adequate and excellent, and equaled 0.76 (95% CI from 0.60 to 0.87) in the test and 0.82 (95% CI from 0.70 to 0.90) in the retest, respectively (see Table 4).

### 3.5. Test–Retest Reliability

Similarly, temporal stability (test–retest reliability) based on the Intraclass Correlation Coefficient (ICC) was excellent for the TMDQ-PL and FQ-PL, and equaled 0.88 (95% CI from 0.75 to 0.95) and 0.96 (95% CI from 0.91 to 0.98), respectively. Regarding the correlation coefficient between the 1 and 2 completion of TMDQ-PL and FQ-PL, significant correlation has been confirmed concerning both TMDQ-PL and FQ-PL results (rs = 0.94 and rs = 0.81, respectively) (see Table 4).

### 3.6. The Inter-Relationships

Both in the test (0.43, *p* = 0.01) and in the retest (0.69, *p* < 0.001), the adapted questionnaires exhibited an inter-correlation (see Table 4).

### 3.7. Comparative Analyses

Table 2 also presents the results of cross-group comparisons for socio-demographic data. In terms of socio-demographic characteristics, there are statistical differences between subgroups among educational levels (*p* = 0.004). The Bonferroni correction revealed that study groups differ statistically in the incidence of having occupational and secondary level of education at (*p* = 0.001). Participants from SG less often had a secondary than occupational level of education, in contrast to participants from HG. The Bonferroni correction also revealed that the study groups differed statistically in terms of having occupational and university levels of education at (*p* = 0.003). Participants from SG less often had university than occupational level of education, in contrast to healthy controls.

There is also a statistical difference between subgroups regarding the place of residence (*p* = 0.001). The Bonferroni correction revealed that the study groups differed statistically in terms of living in the country and a city with over 20,000 inhabitants at *p* = 0.002. More participants from SG lived in the country. A Bonferroni correction also revealed that the study groups differed statistically in terms of living in a city with below 25,000 inhabitants and a city with over 20,000 inhabitants at (*p* = 0.001). Participants from SG lived more often in a city with below 25,000 inhabitants. Results showed that the subgroups statistically differed in terms of working time per week (*p* = 0.013). Patients from SG spend less time on their occupational activity per week. Patients from SG spend fewer hours weekly on an active hobby. A statistical difference between subgroups in marital status (*p* = 0.037) was also revealed. However, a Bonferroni correction showed that certain comparisons between select categories (single, married, divorced, widowed) were statistically insignificant. For details, see Table 2.

Results of comparisons between the TMDQ-PL and FQ-PL between scoliosis patients and healthy controls are presented in Table 5. Regarding TMDQ-PL total score and TMDQ-PL disability level, significant differences at *p* = 0.011 between scoliosis and the healthy group were confirmed.

In particular, significant differences concerning most TMDQ-PL individual questions, such as talking (*p* = 0.023), eating/chewing (*p* = 0.050), singing/playing musical instruments (*p* = 0.002), kissing/hugging (*p* = 0.003), sleeping (*p* = 0.023), effects of any form of treatment (*p* = 0.008), and dizziness (*p* = 0.021), indicating higher levels of pain or discomfort in TMJ everyday function among persons with scoliosis, were discovered.

There was also a statistical difference between both subgroups concerning the FQ-PL total score (*p* < 0.001), revealing more significant complaints regarding TMD among scoliosis patients, compared to healthy controls. For details, see Table 5.

### 3.8. Correlational Analysis by Means of Socio-Demographic Characteristics

Considering TMDQ-PL disability results, a significant correlation was discovered between the level of education and questions concerning talking, brushing teeth/flossing, eating, chewing, and singing or playing musical instruments. Patients with a university education declared higher TMD than those with secondary education (*p* = 0.038, *p* = 0.0002, *p* = 0.032, *p* = 0.019, respectively). Moreover, the working time per week was found to be associated with questions regarding talking, brushing teeth/flossing, and the effects of any form of treatment (rS = −0.42, rS = −0.41 and rS = −0.39, respectively).

Additionally, patients with longer overall working time declared lower pain and discomfort to questions about brushing teeth/flossing and sleeping (rS = −0.37, rS = −0.42).

A significant correlation between the number of children and talking (rS = 0.36) was revealed. We also identified a significant positive correlation between hours of active hobby and tinnitus, or ringing in the ear (patients who spend more hours on active hobbies declared higher tinnitus or ringing in the ear, rS = 0.65). For details, see Table 6.

### 3.9. Associations between Questionnaire Results, Clinical and Radiological Factors

Considering relations between TMDQ-PL and clinical and radiological factors, we identified significant associations between particular variables (apical translation before treatment (rS = 0.52), apical translation after completing the treatment (Rs = 0.39), rib hump angle at the most recent follow-up (rS = 0.36) and talking).

We also identified a significant association between apical translation before treatment and eating/chewing (rS = 0.37).

Moreover, significant correlations were discovered between the Cobb angle after completing the treatment (rS = 0.45), apical translation: after completing the treatment (rS = 0.44), and at the most recent follow-up (rS = 0.46)) and yawning/mouth opening section.

A significant correlation was also revealed between apical translation after completing the treatment and TMDQ-PL total score (rS = 0.36), and also between BMI after completing the treatment and FQ-PL total score (rS = 0.38). For details, see Table 7.

## 4. Discussion

The Milwaukee brace proposed by Blount and Schmidt, known as cervical-thoracic-lumbosacral orthosis (CTLSO), was initially used in the post-operative management of patients with neuromuscular scoliosis, often following polio [32,33]. However, it was adapted relatively quickly for the conservative treatment of idiopathic scoliosis. The specificity of its construction allowed for elongation–extension of the spine between the pelvic girdle and the neck ring in conjunction with derotation.

Over time, the Milwaukee brace became the gold standard in inoperable, conservative treatment of idiopathic scoliosis, especially in the thoracic and thoracolumbar part of the spine [34,35]. Treatment with orthopedic braces is associated with stress, often depressive disorders [20,21]. However, withdrawal from the treatment for a large number of patients was particularly associated with difficulties in adaptation to the Milwaukee brace [36].

At that time, many publications emphasized—apart from the undeniably positive aspects of the brace treatment—the negative ones, including the occurrence of facial deformities, which can be associated with the use of this orthosis [1,2,3,4,37,38].

According to some authors, they could have been caused by the mechanical pressure of the neck ring on craniofacial structures in patients during the growth spurt, which is also the period with highest risk of scoliosis progression. It does not change the fact that the literature has long emphasized the increased frequency of malocclusion in patients with scoliosis, which cannot be related exclusively to orthopedic treatment using the CTLSO-type brace [5,6,8].

This is confirmed by Huggare et al., who evaluated 22 patients aged 12 to 34 treated with a Boston-type brace that does not involve prolonged pressure within the mandible area, as in the case of a Milwaukee brace having a neck brace [8]. In the analyzed group, these authors reported abnormalities in the position of the skull in relation to the cervical spine, and abnormalities in the mandibular, occlusal, and orbital planes. They also described a higher incidence of lateral malocclusions [8].

Among the works describing disorders in the craniofacial region in patients with scoliosis, the study by Prager et al. should also be mentioned as it assessed 63 patients who had been treated for scoliosis as well as other postural abnormalities not entirely defined by them. The authors describe an increased incidence of different dentoalveolar anomalies in the evaluated population. Among the most common anomalies, they listed crossbite [39]. These reports are consistent with the results of Korbmacher et al., in which the authors assessed a group of 55 patients with unilateral crossbite. The authors observed a statistically significant higher frequency of occurrence of unilateral crossbite in patients with scoliosis [40]. The assessment of the scale of this problem was also carried out by Segatto et al. [41]. In studies performed among the pediatric population with spinal deformities, they examined the coexistence of malocclusion in 28 patients with scoliosis. The control group consisted of 68 orthopedically healthy children. In the orthodontic study, the authors included occlusal relations in the sagittal, vertical, and transverse planes, as well as TMD. On the basis of a clinical trial, the authors recognized TMD in almost 25% of patients with scoliosis. In addition, based on the analysis of cephalometric photographs in the group of patients with scoliosis, they noted an increased incidence of mandibular retrusion as well as protrusion within the jaw [41]. In comparison with the control group, patients with scoliosis reported more frequent occlusion disorders in the form of midline deviation. They also observed a statistically significant smaller range of mandibular lateral movement, a problem that affected approximately 50% of patients with postural scoliosis [41]. In turn, studies by Muhlbach and Rink found the occurrence of various disorders in the bite without detailed reference to the type of defect. These disorders are found in more than 80% of the examined patients with scoliosis [42]. Also, in the slightly newer studies conducted on a very large sample of 600 patients with scoliosis as well as other defects in posture, Komakhidze assessed the frequency of dentofacial anomalies at 70.2%. However, in the control group in which no postural defects were found in the clinical trial, the presence of such disorders was recognized in 41% of the examined persons [43].

Returning, however, to the main thread about the impact of the Milwaukee brace on the growth and development of the facial skeleton, it should be noted that according to these authors, the brace most often caused a protrusion of anterior teeth, intrusion of posterior teeth, and loss of facial height associated with the inhibition of maxillary and mandibular growth [32].

The occurrence of these changes, according to Wolff’s law, was to be the result of a long-lasting multiyear period of about 22–23 h per day of pressure, with a strength of about 4 pounds per jaw, caused by the neck ring [2,3].

As a consequence, it led to disturbed ossification, while the blood supply in this area was decreased. [3]. Significantly, the changes in facial proportions were visible after six months of using the Milwaukee brace, and coexisted with disorders in electromyographic muscle activity [44].

Increasing knowledge about the type and rate of development of mandibular developmental disorders following the brace treatment resulted in the creation of various types of positioners used, among others, by Olin et al. or Alexander [1,3].

Alexander evaluated 14 patients treated with the Milwaukee brace. After dividing them into two groups of seven people each, he used a positioner to protect the occlusion (positioner type o appliance) in one of them [3]. In the second group, he did not use the positioner, and subjected it to observation only. In the assessment, detailed cephalometric photographs were analyzed. Among the observations made, it was found that brace treatment has a significant impact on the growth within the lower part of the craniofacial region. The author noted the occurrence of such anomalies as reduction in the total anterior face height, lift of palatal plane, with a tendency to flatten the palatal vault. In the mandible area, an increase in the body structure at the mandibular angle was described. Within the bite itself, the author found the occurrence of extruding of incisors and depressing of molars [2]. These observations are consistent with reports by Rock and Baker [38]. These authors evaluated 25 patients aged 5 to 19 years treated with the Milwaukee brace for an average of 2.5 years. The evaluation was based on side images of the skull, in accordance with which parameters such as total face height, upper face height, lower face height, upper and lower dental height, and maxilla length were determined. In the opinion of Rock and Baker, brace treatment contributes to the reduction in total craniofacial height compared to the control group not treated for scoliosis [38]. In turn, in the data provided by Dayan et al., the authors also observed a reduction in the total vertical dimension of the craniofacial region, compared to the control group, as well as increased protrusion within the mandible and jaw [45]. In the work of Luedtke, the author discusses in detail the issues related to the orthodontic treatment of a patient during brace treatment. Based on the evaluation of 10 patients treated for one year, the author did not find any significant disturbances associated with the reduction in the total height of the face [4].

A separate issue that needs to be discussed is the mechanism of overload-degenerative changes in the temporomandibular joint following the use of the Milwaukee brace, which was described in detail in Pollack’s work [37]. According to this author, during a multi-month cycle of brace treatment, the temporomandibular joint is exposed to the effect of upward force against the interior border of the mandible. This force far exceeds the values used in orthodontic treatment during the correction of malocclusion, and in addition, its impact increases during the patient’s sleep. In the author’s opinion, too much pressure leads to rotation of the mandible around the temporomandibular joint into an overclosed position. Reducing the vertical dimension of the craniofacial region leads to secondary crowding of the incisors, which in turn leads to their protrusion. In the case of lateral teeth, they deflect in the direction of the reduced resistance. At the same time, the secondary reduction in the space for the tongue results in a further displacement of the incisors in the proximal direction, and the molar teeth in the collateral direction. [37] In turn, according to Bunch, the pressure exerted by the brace affects the position of the whole denture in relation to the cranial base and supporting bone [46]. Another negative consequence of using a brace is the reduction in posterior face height, which Alexander notes in his research [2]. This is most likely due to the superior intrusion of the condylar head into the glenoid fossa. This intrusion and overclosure, combined with the occurrence of increased pressure within the TMJ cartilage, can cause premature degenerative changes within the joint.

The problem that remains the most difficult to explain is the effect of scoliosis and previous treatment on TMD. This is because the Milwaukee brace is currently used only sporadically in the treatment of idiopathic scoliosis, having been effectively replaced by a TLSO brace. Hence the small number of publications on this subject [9,10,11].

The second problem is the interdisciplinary dimension of ailments located in the TMJ, which results in patients with these problems being treated by neurologists, dentists, and orthopedists.

In our study, we have assumed that socio-demographic characteristics differ significantly between scoliotic and non-scoliotic study samples. We found statistical differences between subgroups in areas such as educational level, place of residence, working time per week, and marital status. Considering the differences in subgroups, we should take into account that factors related to living environments can influence the patients’ condition.

The results of our study show clearly that TMD was a significant problem among the group of scoliotic patients compared to healthy controls. We discovered a statistically significant difference between TMDQ-PL and FQ-PL in the total score, and answers to questions about talking, eating/chewing, singing/playing musical instruments, kissing/hugging, sleeping, effect of any form of treatment, and dizziness. Moreover, results of the TMDQ-PL revealed that the most disturbing symptom of TMJ in scoliosis is dizziness. Furthermore, it was revealed that scoliotic patients indicate higher levels of disability. The aforementioned results proved our primary hypothesis.

Interestingly, our study revealed a significant relation between the level of education and TMDQ-PL results. Patients with a university education declared higher TMD than those with secondary education.

Among the studies assessing the impact of the use of the Milwaukee brace on the temporomandibular joint with a slightly longer period of observation, the one conducted by Hess et al. deserves attention. The authors assessed a group of patients treated for scoliosis with a Milwaukee brace with a minimum follow-up of 20 years after the end of the orthosis use. Noting that the growth of the mandible lasts more than a year after the growth spurt of the whole organism, the authors found the appearance of compensating mechanisms in the craniofacial region after the brace was discontinued. In the short 5-year period, they did not find any significant pain complaints regarding TMJ in the analyzed group of patients [11].

From the point of view of functional ailments within TMJ, the presence of scoliosis with small and medium angular values is, according to some authors, including Yongnam and Youngsook, the reason for the occurrence of temporomandibular joint mobility disorders. And thus, in the work where they evaluated 31 patients with diagnosed scoliosis, 19 participants underwent therapeutic exercises for eight weeks while the others did not perform exercises. The authors noted an increase in the range of motion of the temporomandibular joint in persons performing exercises in relation to the control group. In addition, they found that the greater the reduction in the range of joint mobility, the higher were the Cobb angle values found among the analyzed patients [10]. These reports coincide with the opinions of Friedman and Weisberg, who believed that muscle balance disorders in the temporomandibular joints underlie abnormalities in the mastication process, and may also lead to secondary dentofacial anomalies [47]. Other authors who emphasize the problem of balancing the distribution of muscle strength include Oekson, who claimed that abnormalities in the position of the mandible testify to the deterioration of masticatory muscles. These anomalies in the subsequent stages of the disease development may be the cause of the deterioration of the efficiency of the entire stomatognathic system [48].

Summing up the results of our research, it should be acknowledged that they confirm the well-known fact that a medicine, in this case a brace, used in good faith may reveal negative effects on organs far removed from the scoliosis nucleus in a distant study.

In addition, our research showed excellent internal consistency of the Polish version of TMDQ-PL and FQ-PL for the whole study group. Analyses of the temporal stability (test–retest reliability) and content validity confirmed that TMDQ-PL and FQ-PL have excellent reliability and good concurrent validity. Moreover, we confirmed an inter-correlation, both in the test and the retest. The Polish versions of TMDQ-PL and FQ-PL meet methodological criteria, and are useful tools for international comparative studies of TMD in scoliotic patients.

### 4.1. Study Limitations

Our study reveals several limitations. Firstly, our study cannot be compared to many earlier studies because there is a paucity of previous research assessing the effects of the Milwaukee brace on TMD in scoliotic patients. Secondly, we included only 30 AIS patients in the patient sample (*n* = 30). Thirdly, it is impossible to make conclusions about causal relationships between selected variables based on a correlational study.

### 4.2. General Implications for Clinical Practice

Taken together, the results of our study provide clinicians with reliable data concerning TMD in the long-term follow-up after completing a Milwaukee brace treatment. We believe this may lead to increasing the effectiveness of therapy outcomes. 

## 5. Conclusions

Adult patients with scoliosis treated conservatively present limitations in everyday activities connected with TMJ. It was revealed in the variety of curve-related factors in a long-term follow-up of wearing Milwaukee brace that influence TMJ. Our study confirms that it is essential to include orthodontists’ practice in the holistic treatment of AIS. Another important learning point in our work is that TMDQ-PL and FQ-PL are reliable tools comparable with their original versions, and can be recommended for use in international comparative studies of TMD in scoliosis patients.

## Figures and Tables

**Table 1:** Clinical characteristics of patients.

Characteristics	Mean (SD)	Range	*n* (%)
Brace application (hours/day)	22.9 (0.31)	22–23	---
Brace application (months)	45.47 (20.00)	24–104	---
Time after completed treatment (years)	27.77 (3.30)	23–35	---
Body Mass Index			---
Before treatment	17.47 (3.78)	13.64–22.83	---
After completed treatment	19.91 (2.57)	15.81–25.24	---
In the present study	24.03 (4.05)	17.78–34.34	---
Curve type			
Thoracic	---	---	21 (70.0)
Thoraco-lumbar	---	---	2 (6.67)
Lumbar	---	---	7 (23.33)
Curve size of the major curve (Cobb angle)			
Before treatment	32.2 (5.59)	20–40	---
After completed treatment	37.87 (12.75)	10–70	---
In the present study	45.03 (17.41)	10–97	---
Curve change from end of treatment to present study	7.16 (7.64)	0–27	---
Apical translation (cm) *			
Before treatment	2.05 (0.98)	0.2–4	---
After completed treatment	2.65 (1.30)	0.3–5.1	---
In the present study	3.66 (1.99)	0.5–9.4	---
Rib hump angle in present study	10.37 (4.20)	4–20	---
Rib hump height in present study (cm)	3.33 (1.53)	1–7	---
Thoracic kyphosis in present study (angle)	25.33 (11.06)	9–62	---
Lumbar lordosis in present study (angle)	54.63 (10.59)	27–76	---

Note * the degree of the apical translation of center sacral vertical line (CSVL) according to the Harms Study Group; standard deviation (SD).

**Table 2:** Socio-demographic characteristics of patients and healthy controls.

Characteristics	Patient Group			Healthy Controls			*p* Value
	Mean (SD)	Range	*n* (%)	Mean (SD)	Range	*n* (%)	
Age at the start of treatment (yrs.)	12.43 (1.83)	10–14	---	Not applicable			
Age in present study (yrs.)	41.13 (3.87)	35–50	---	42.05 (7.41)	22–61	----	*p* = 0.326
Marital status							*p* = 0.037 *
Single	---	---	1 (3.33)	---	---	8 (19.0)	
Married	---	---	28 (93.34)	---	---	29 (69.00)	
Divorced	---	---	1 (3.33)	---	---	5 (11.9)	
Widowed	---	---	0 (0)	---	---	0 (0)	
Educational level							*p* = 0.004 *
Elementary	---	---	2 (6.67)	---	---	2 (4.8)	
Occupational	---	---	7 (23.33)	---	---	0 (0)	
Secondary	---	---	7 (23.33)	---	---	19 (45.2)	
University	---	---	14 (46.67)	---	---	21 (50)	
Place of residence							*p* = 0.001 *
Country	---	---	12 (40.0)	---	---	8 (19.0)	
Below 25,000	---	---	12 (40.0)	---	---	7 (16.7)	
Between 25,000 and 20,000	---	---	2 (6.67)	---	---	4 (9.5)	
Over 20,000	---	---	4 (13.33)	---	---	23 (54.8)	
Working time per week (hours)	33.21 (15.41)	0–60	---	42.88 (13.96)	5–70	---	*p* = 0.013 *
Overall working time (yrs.)	17.37 (19.13)	0–30	---	19.55 (8.94)	1–40	---	*p* = 0.317
Active hobby	---	---	13 (43.33)	---	---	16 (38.1)	*p* = 0.656
Active hobby per week (hours) ***	3.46 (3.78)	1–15	---	5.12 (4.30)	2–20	---	*p* = 0.038 *

Note * *p* < 0.05; *** out of 13 patients and 17 healthy controls with an active hobby; standard deviation (SD).

**Table 3:** Descriptive statistics of the TMDQ-PL and FQ-PL in scoliosis patients.

Questionnaire	Scoliosis Patients											
	Test						Retest					
	Mean	Min	Max	95% CI		SD	Mean	Min	Max	95% CI		SD
				From	To					From	To	
TMDQ-PL												
Ouestion 1	0.37	0	2	0.14	0.60	0.61	0.30	0	2	0.10	0.50	0.53
Question 2	0.13	0	2	−0.03	0.30	0.43	0.07	0	1	-0.03	0.16	0.25
Ouestion 3	0.27	0	2	0.07	0.46	0.52	0.20	0	1	0.05	0.35	0.40
Ouestion 4	0.60	0	3	0.25	0.95	0.93	0.37	0	2	0.12	0.62	0.66
Ouestion 5	0.30	0	3	0.04	0.56	0.70	0.30	0	3	0.06	0.54	0.65
Ouestion 6	0.47	0	3	0.16	0.77	0.81	0.23	0	2	0.05	0.42	0.50
Ouestion 7	0.30	0	2	0.10	0.50	0.53	0.27	0	1	0.10	0.43	0.44
Ouestion 8	0.77	0	3	0.40	1.13	0.97	0.53	0	3	0.23	0.84	0.81
Ouestion 9	0.23	0	1	0.07	0.39	0.43	0.17	0	1	0.03	0.31	0.37
Ouestion 10	0.83	0	3	0.49	1.17	0.91	0.80	0	2	0.50	1.10	0.80
TMDQ-PL total score	4.27	0	16	2.44	6.10	4.89	3.23	0	14	1.79	4.68	3.86
TMDQ-PL level of disability (%)	11.0	0	40	0.06	0.15	12.0	8.0	0	30	0.04	0.12	9.0
FQ-PL total score	32.67	5	80	25.22	40.11	19.94	32	0	85	23.73	40.27	22.15

Note: Question 1 is Talking; question 2 is Brushing Teeth/Flossing; question 3 is Eating, Chewing; question 4 is Singing, Playing Musical Instruments; question 5 is Yawning, Mouth Opening; question 6 is Kissing, Hugging; question 7 is Sleeping; question 8 is Effects of Any Form of Treatment; question 9 is Tinnitus; question 10 is Dizziness.; TMD Disability Index Questionnaire; Fonseca Questionnaire; standard deviation (Mean ± SD); confidence intervals (CI).

**Table 4:** Psychometric properties of the TMDQ-PL and FQ-PL.

	Test		Retest		Test–Retest Reliability			Correlation between TMDQ-PL and FQ-PL
	Cronbach’s Alpha	95% Confidence Intervals	Cronbach’s Alpha	95% Confidence Intervals	Intraclass Correlation Coefficient	95% Confidence Intervals	Correlation between TMDQ-PL in Test and Retest and between FQ-PL in Test and Retest	
TMDQ-PL total score	0.87	0.79–0.93	0.87	0.79–0.93	0.88	0.75–0.95	rS = 0.94 *p* = 0.000	test rS = 0.43 *p* = 0.01
FQ-PL total score	0.76	0.60–0.87	0.82	0.70–0.90	0.96	0.91–0.98	rS = 0.81 *p* = 0.000	retest rS = 0.69 *p* < 0.001

Psychometric properties of the TMDQ-PL and FQ-PL-preliminary validation. Floor and ceiling effects (content validity). Regarding TMDQ-PL total score, the floor effect can be seen in 8 patients (26.67%) in the test and in 9 patients (30%) in the retest. Concerning FQ-PL, there was no floor effect in the test, whereas it can be seen in the retest in 4 patients (13.33%). We did not discover any ceiling effects among TMDQ-PL and FQ-PL total scores.

**Table 5:** Descriptive statistics of the TMDQ-PL and FQ-PL in healthy controls, and results of comparisons between scoliosis patients and healthy controls.

Questionnaire	Healthy Controls						Results of Comparisons between Scoliosis Patients and Healthy Controls *p* Value
	Test						
	Mean	Min	Max	95%CI		SD	
				From	To		
TMDQ-PL							
Ouestion 1	0.10	0	1	0	0.19	0.30	*p* = 0.023 *
Ouestion 2	0.07	0	2	−0.04	0.18	0.34	*p* = 0.407
Ouestion 3	0.07	0	1	−0.01	0.15	0.26	*p* = 0.050 *
Ouestion 4	0.10	0	2	−0.02	0.21	0.37	*p* = 0.002 *
Ouestion 5	0.07	0	1	−0.01	0.15	0.26	*p* = 0.096
Ouestion 6	0.05	0	1	−0.02	0.11	0.22	*p* = 0.003 *
Ouestion 7	0.07	0	1	−0.01	0.15	0.26	*p* = 0.023 *
Ouestion 8	0.24	0	2	0.07	0.40	0.53	*p* = 0.008 *
Ouestion 9	0.26	0	2	0.11	0.42	0.50	*p* = 0.926
Ouestion 10	0.38	0	2	0.19	0.58	0.62	*p* = 0.021 *
TMD-PL total score	1.40	0.82	1.99	0.82	1.99	1.89	*p* = 0.011 *
TMD-PL level of disability (%)	0.04	0	0.25	0.02	0.05	0.05	*p* = 0.011 *
FQ-PL total score	14.64	0	45	11.16	18.13	11.17	*p* <0.001 *

Note: Question 1 is Talking; question 2 is Brushing Teeth/Flossing; question 3 is Eating, Chewing; question 4 is Singing, Playing Musical Instruments; question 5 is Yawning, Mouth Opening; question 6 is Kissing, Hugging; question 7 is Sleeping; question 8 is Effect of Any Form of Treatment; question 9 is Tinnitus; question 10 is Dizziness. * *p* < 0.05; TMD Disability Index Questionnaire; Fonseca Questionnaire; standard deviation (Mean ± SD); confidence intervals (CI).

**Table 6:** Associations between the socio-demographic data, TMD Disability Index, and Fonseca results.

Questionnaires	Age at the Present (yrs)	Age at the Start of Treatment (yrs)	Marital Status	Educational Level	Place of Residence	Working Time per Week (Hours)	Overall Working Time (yrs)	No. of Children	Active Hobby per Week (Hours)
TMDQ-PL									
Question 1	rS = −0.07	rS = 0.08	Not applicable	*p* = 0.038 *	*p* = 0.640	rS = −0.42 *	rS = −0.14	rS = 0.36 *	rS = 0.09
Question 2	rS = −0.19	rS = −0.11		*p* = 0.0002 *	*p* = 0.726	rS = −0.41 *	rS = −0.37 *	rS = 0.20	rS = 0.02
Question 3	rS = −0.05	rS = −0.13		*p* = 0.032 *	*p* = 0.511	rS = −0.30	rSv0.13	rS = 0.16	rS = 0.20
Question 4	rS = −0.16	rS = −0.20		*p* = 0.019 *	*p* = 0.987	rS = −0.36	rS = −0.31	rS = 0.30	rS = −0.33
Question 5	rS = −0.05	rS = −0.27		*p* = 0.731	*p* = 0.608	rS = −0.19	rS = −0.04	rS = 0.04	rS = 0.43
Question 6	rS = −0.06	rS = 0.004		*p* = 0.635	*p* = 0.668	rS = −0.03	rS = −0.25	rS = −0.06	rS = 0.10
Question 7	rS = −0.15	rS = −0.04		*p* = 0.075	*p* = 0.453	rS = −0.28	rS = −0.42 *	rS = 0.28	rS = −0.02
Question 8	rS = −0.22	rS = −0.10		*p* = 0.165	*p* = 0.743	rS = −0.39 *	rS = −0.29	rS = 0.14	rS = −0.31
Question 9	rS = −0.15	rS = −0.11		*p* = 0.572	*p* = 0.576	rS = 0.01	rS = −0.21	rS = −0.10	rS = 0.65 *
Question 10	rS = −0.24	rS = −0.30		*p* = 0.947	*p* = 0.916	rS = 0.11	rS = −0.16	rS = −0.23	rS = 0.17
TMDQ-PL total score	rS = −0.15	rS = −0.21		*p* = 0.177	*p* = 0.692	rS = −0.28	rS = −0.26	rS = 0.09	rS = 0.09
FQ-PL total score	rS = −0.24	rS = −0.07		*p* = 0.628	*p* = 0.807	rS = 0.02	rS = −0.17	rS = −0.17	rS = 0.26

Note: Question 1 is Talking; question 2 is Brushing Teeth/Flossing; question 3 is Eating, Chewing; question 4 is Singing, Playing Musical Instruments; question 5 is Yawning, Mouth Opening; question 6 is Kissing, Hugging; question 7 is Sleeping; question 8 is Effect of Any Form of Treatment; question 9 is Tinnitus; question 10 is Dizziness. * *p* < 0.05; TMD Disability Index Questionnaire; Fonseca Questionnaire; standard deviation (Mean ± SD); confidence intervals (CI).

**Table 7:** Correlational analysis between clinical and radiological patient characteristics and TMD Disability Index and Fonseca results.

	Question 1	Question 2	Question 3	Question 4	Question 5	Question 6	Question 7	Question 8	Question 9	Question 10	TMDQ-PL Total Score	FQ-PL Total Score
Brace application (hours/day)	Not applicable											
Brace application (months)	rS = 0.08	rS = 0.14	rS = 0.09	rS = 0.09	rS = −0.11	rS = 0.06	rS = −0.09	rS = 0.11	rS = 0.05	rS = 0.23	rS = 0.17	rS = −0.18
Follow-up after completing the treatment (years)	rS = 0.11	rS = 0.25	rS = 0.02	rS = 0.16	rS = 0.05	rS = 0.03	rS = −0.09	rS = −0.24	rS = 0.08	rS = −0.05	rS = −0.01	rS = −0.23
Body Mass Index												
Before the treatment	rS = 0.16	rS = −0.11	rS = 0.10	rS = −0.25	rS = −0.18	rS = −0.12	rS = −0.14	rS = −0.24	rS = −0.17	rS = −0.09	rS = −0.18	rS = −0.14
After completing the treatment	rS = −0.09	rS = −0.09	rS = −0.05	rS = −0.16	rS = −0.15	rS = −0.13	rS = −0.18	rS = −0.27	rS = 0.06	rS = −0.09	rS = −0.21	rS = −0.38 *
At the most recent follow-up	rS = −0.18	rS = 0	rS = 0.07	rS = −0.33	rS = 0.01	rS = −0.03	rS = −0.18	rS = −0.29	rS = 0.05	rS = −0.02	rS = −0.20	rS = 0.05
Curve type	*p* = 0.920	*p* = 0.323	*p* = 0.622	*p* = 0.161	*p* = 0.767	*p* = 0.815	*p* = 0.479	*p* = 0.700	*p* = 0.628	*p* = 0.511	*p* = 0.294	*p* = 0.829
Curve size of the major curve (Cobb angle) **												
Before the treatment	rS = 0.21	rS = 0.23	rS = 0.21	rS = 0.28	rS = 0.15	rS = −0.03	rS = 0.04	rS = 0.15	rS = −0.36	rS = −0.27	rS = 0.03	rS = 0.02
After completing the treatment	rS = 0.09	rS = 0.08	rS = 0.22	rS = 0.17	rS = 0.45 *	rS = 0.16	rS = −0.01	rS = 0.06	rS = −0.05	rS = 0.16	rS = 0.18	rS = 0.09
At the most recent follow-up	rS = 0.17	rS = 0.10	rS = 0.19	rS = 0.23	rS = 0.36	rS = 0.15	rS = 0	rS = 0.14	rS = −0.19	rS = 0.20	rS = 0.20	rS = 0.09
Cobb angle in brace	rS = −0.10	rS = −0.17	rS = −0.15	rS = −0.01	rS = −0.02	rS = −0.03	rS = −0.19	rS = −0.06	rS = −0.34	rS = −0.09	rS = −0.13	rS = 0.06
Apical translation (cm) ***												
Before the treatment	rS = 0.52 *	rS = 0.17	rS = 0.37 *	rS = 0.23	rS = 0.16	rS = 0	rS = 0.03	rS = 0.18	rS = −0.09	rS = −0.12	rS = 0.20	rS = 0.02
After completing the treatment	rS = 0.39 *	rS = 0.19	rS = 0.30	rS = 0.36	rS = 0.44 *	rS = 0.22	rS = 0.17	rS = 0.32	rS = 0.11	rS = 0.22	rS = 0.36 *	rS = 0.30
At the most recent follow-up	rS = 0.24	rS = 0.17	rS = 0.30	rS = 0.29	rS = 0.46 *	rS = 0.14	rS = 0.02	rS = 0.17	rS = −0.004	rS = 0.16	rS = 0.28	rS = 0.04
Rib hump angle at the most recent follow-up	rS = 0.36 *	rS = 0.24	rS = 0.20	rS = 0.23	rS = 0.06	rS = −0.03	rS = −0.04	rS = 0.03	rS = −0.23	rS = −0.12	rS = 0.10	rS = 0.05
Rib hump height at the most recent follow-up (cm)	rS = 0.29	rS = 0.15	rS = 0.12	rS = 0.09	rS = 0.01	rS = −0.04	rS = −0.12	rS = −0.04	rS = 0	rS = −0.21	rS = 0.02	rS = −0.01
Thoracic kyphosis at the most recent follow-up (angle)	rS = 0.14	rS = 0.11	rS = 0.13	rS = 0.17	rS = −0.05	rS = 0.09	rS = 0.02	rS = 0.08	rS = −0.05	rS=-0.16	rS = 0.13	rS = 0
Lumbar lordosis at the most recent follow-up (angle)	rS = 0.08	rS = 0.17	rS = 0.05	rS = 0.17	rS = 0.04	rS = −0.09	rS = −0.08	rS = 0.02	rS = −0.04	rS = −0.22	rS = 0.11	rS = 0
−Vital capacity (VC) (cm^3^)												
Before the treatment	rS = 0.25	rS = 0.05	rS = 0.19	rS = −0.18	rS = −0.09	rS = −0.02	rS = −0.11	rS = −0.14	rS = −0.33	rS = −0.08	rS = −0.10	rS = −0.05
After completing the treatment	rS = 0.13	rS = −0.04	rS = 0.02	rS = −0,13	rS = −0,21	rS = 0,05	rS = −0.06	rS = 0.01	rS = −0.14	rS = 0.09	rS = 0.07	rS = 0.05
At the most recent follow-up	rS = 0.01	rS = 0.06	rS = 0.12	rS = −0.08	rS = −0.02	rS = −0.03	rS = 0.05	rS = 0.01	rS = 0.08	rS = 0.02	rS = −0.06	rS = 0.11

Note: Question 1 is Talking; question 2 is Brushing Teeth/Flossing; question 3 is Eating, Chewing; question 4 is Singing, Playing Musical Instruments; question 5 is Yawning, Mouth Opening; question 6 is Kissing, Hugging; question 7 is Sleeping; question 8 is Effect of Any Form of Treatment; question 9 is Tinnitus; question 10 is Dizziness; * *p* < 0.05; ** according to Nachemson and Peterson, successful treatment was defined as an increase in curvature of less than 6° from the start of bracing; *** the degree of apical translation of the center sacral vertical line (CSVL) according to the Harms Study Group; TMD Disability Index Questionnaire; Fonseca Questionnaire.

## Data Availability

The data presented in this study are available on request from the corresponding author. The data are not publicly available due to privacy.
